# C-arm cone-beam computed tomography-guided minimally invasive open excision of an osteoid osteoma undetectable on fluoroscopy: A case report

**DOI:** 10.1016/j.ijscr.2019.06.062

**Published:** 2019-07-08

**Authors:** Takashi Higuchi, Norio Yamamoto, Katsuhiro Hayashi, Akihiko Takeuchi, Kensaku Abe, Yuta Taniguchi, Yoshihiro Araki, Hiroyuki Tsuchiya

**Affiliations:** Department of Orthopaedic Surgery, Graduate School of Medical Sciences, Kanazawa University, 13-1 Takara-machi, Kanazawa, 920-8641, Japan

**Keywords:** Osteoid osteoma, A robotic C-arm cone-beam computed tomography, Minimally invasive surgery, Case report

## Abstract

**Introduction:**

The small size of a nidus and the extensive sclerosis around it often make it difficult to identify tumors from the surrounding normal bone by fluoroscopy, and a large amount of bone resection might be required to secure complete removal during osteoid osteoma surgery. In this report, we present the first case of radiographically invisible osteoid osteoma that was successfully resected under a robotic C-arm cone-beam computed tomography (CBCT) imaging system.

**Presentation of case:**

A 14-year-old boy presented with persistent pain around the left knee joint was diagnosed with osteoid osteoma of the left distal femur. Since the tumor was not detectable by intraoperative fluoroscopy, a CBCT system was used for guidance during an open tumor resection. For a less invasive surgery, we repurposed the dilator and retractor of the microendoscopic discectomy (MED) system and resected the tumor using the wider trephine tips for bone biopsy. At the final follow-up one year later, the function of the afflicted limb was normal with no complaint of any symptoms. Plain radiograph at the final follow-up showed new bone formation completely filling the bone defect, without local recurrence.

**Discussion:**

CBCT allowed for clear intraoperative visualization of the osteoid osteoma, thereby promising a secure resection and less invasive osteoid osteoma treatment. Furthermore, the repurposed use of the dilator and retractor of the MED system as well as the wider trephine tips for bone biopsy made the procedure less invasive.

**Conclusion:**

CBCT-guided minimally invasive open excision surgery could be a useful option for the management of radiographically invisible osteoid osteoma.

## Highlights

• A robotic C-arm cone-beam computed tomography (CBCT) system provides a clear intraoperative visualization of the osteoid osteoma.
• C-arm CBCT allowed precise access to the tumor, accurate tumor resection, and minimal sacrifice of the normal bone in osteoid osteoma surgery.
• Repurpose use of the dilator, retractor, and wider trephine tips achieved more conservative results with a smaller skin incision.

## 1 Introduction

Osteoid osteoma is a benign bone tumor that occurs in children and adolescents, and causes severe pain [1,2]. Osteoid osteoma is sometimes missed on plain radiography because it is usually small; it could be ≤1 cm in size, and the nidus or periosteal reaction might not always be radiographically visible [1,3]. Surgical resection is still the standard treatment for osteoid osteoma and often performed under fluoroscopic guidance [4,5]. We report a radiographically invisible osteoid osteoma of the distal femur that was successfully resected under robotic C-arm cone-beam computed tomography (CBCT), which is a multi-axis C-arm radiography system and provides CT-like images intraoperatively. Furthermore, by repurposing the dilator and retractor of the microendoscopic discectomy (MED) system and using the wider trephine tips for bone biopsy, a less invasive surgery with a small incision and minimal soft-tissue damage was possible. This report fulfills the SCARE criteria [6].

## 2 Presentation of case

**Fig. 1:**
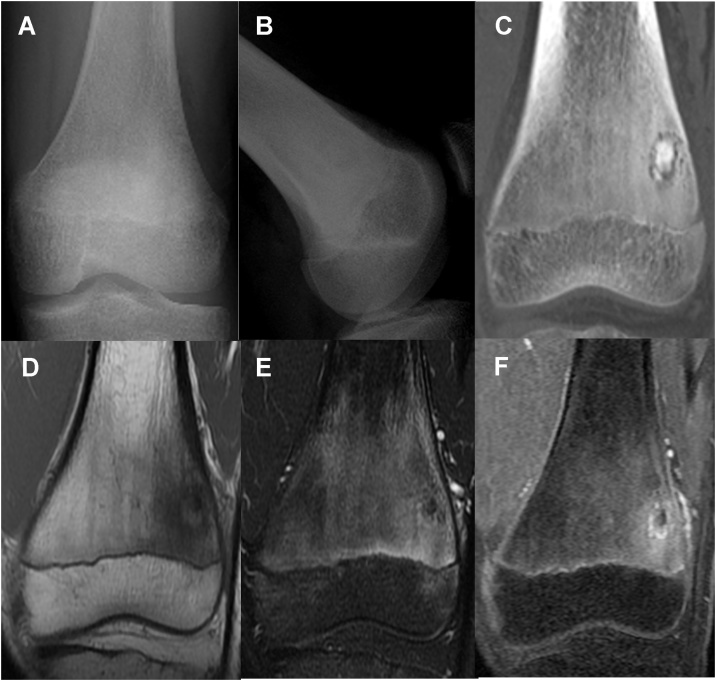
(A) Plain radiography of the left distal femur. The nidus was undetectable on the frontal view. (B) Mild radiolucent area with slight central calcification was detected on the lateral view. (C) Computed tomography coronal view (bone condition). (D) Coronal T1-weighted image. (E) Coronal T2-weighted image with fat suppression. (F) Coronal Gadolinium contrast image.

**Fig. 2:**
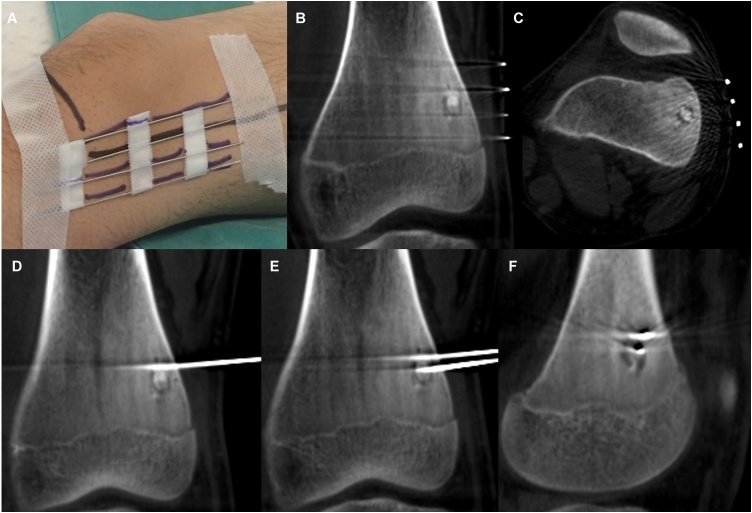
(A) An instant marker was set on the lateral side of the knee. (B) Confirmation of tumor location with cone-beam computed tomography coronal images. (C) Axial image. (D) First attempt. The Kirschner wire was inserted proximal to the tumor. (E) Second attempt using wire sleeves for parallel insertion. The Kirschner wire was inserted to the center of the tumor. (F) Sagittal image.

**Fig. 3:**
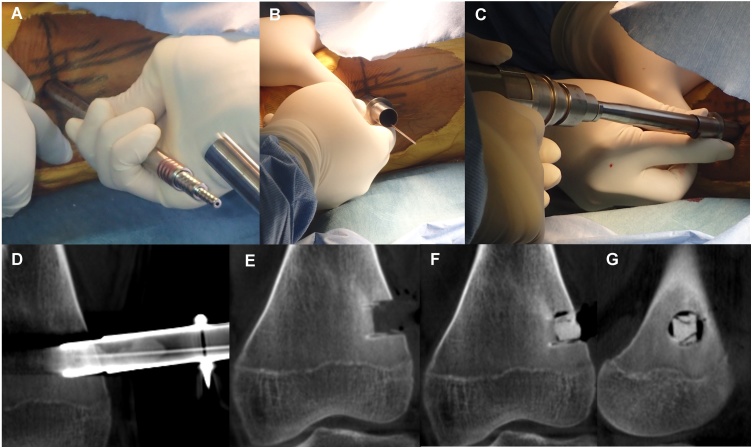
(A) The soft-tissue was dilated with the dilator from the microendoscopic discectomy (MED) system. (B) Retraction with the MED retractor. (C) Drilling with the 120 mm trephine tip. (D) Confirmation of the tumor in the drilled route. (E) Tumor bone resection. (F) Insertion of a β-tricalcium phosphate block into the bone defect. Coronal cone-beam computed tomography (CBCT) image. (G) Sagittal CBCT image.

**Fig. 4:**
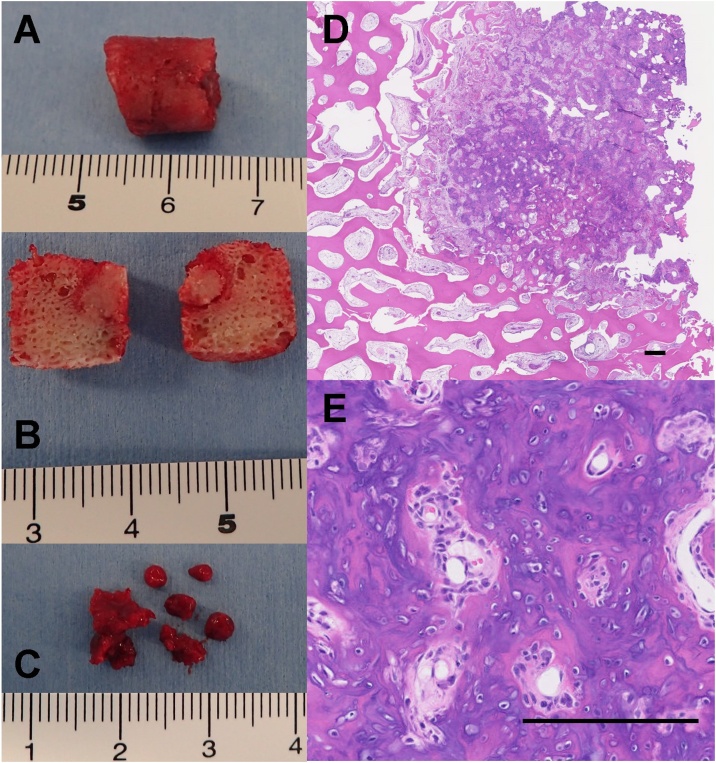
(A) Resected bone. (B) Vertical split of the tumor bone. (C) Additional curetted bone. No tumor tissue was detected. (D, E) Hematoxylin and eosin staining. Scale bars indicate 100 μm. The nidus included anastomosing trabeculae of the osteoid and woven bone.

**Fig. 5:**
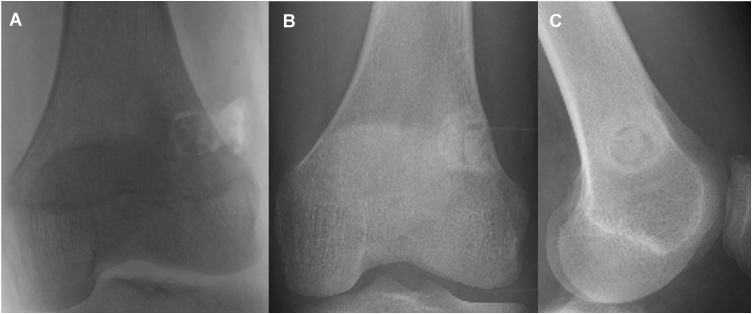
(A) Fluoroscopic image of cone-beam computed tomography immediate after the surgery. (B, C) Radiographs of the final follow-up. The bone defect was completely filled by new bone.

A 14-year-old boy was referred to our hospital because of persistent pain around the left knee joint since approximately 3 months before. A plain radiography revealed, though extremely difficult to discern, a small, relatively radiolucent area with a tiny central calcification in the distal lateral femur (Fig. 1A and B). This was clearly visible as an approximately 1 cm oval nidus, including a mineralized osteoid in the center, on CT scan (Fig. 1C). Magnetic resonance imaging (MRI) revealed a low-signal oval lesion that reflected the osteoid on T1-weighted imaging (Fig. 1D), and a surrounding high-signal area on T2-weighted imaging that reflected the adjacent bone marrow edema (Fig. 1E). Only the nidus was well-enhanced on contrast MRI, which might indicate the hypervascularity of the nidus (Fig. 1F). As the tumor was not detectable on standard intraoperative fluoroscopy, a robotic C-arm CBCT system (Artis zeego, Siemens AG, Forchheim, Germany), was used for guidance during open resection of the tumor. An incision line was marked on the skin above the tumor under CBCT imaging guidance by using an instant marker made by arranging Kirschner wires at 1 cm intervals (Fig. 2A, B, and C). An approximately 1 cm skin incision was made, and a Kirschner wire was inserted toward the tumor, with CBCT guidance at each step (Fig. 2D, E, and F). The soft-tissue was dilated bluntly in small parts by using the dilator originally used for MED surgery to minimize soft-tissue damage (Fig. 3A). After exposing the lateral side of the femur, 120 mm trephine tip for bone biopsy was drilled according to the MED retractor (Fig. 3B and C). After confirming on the CBCT image that the tumor was in the drilled route (Fig. 3D), the tumor-bearing bone was resected *en-bloc* with the trephine tip and additional use of a round chisel (Fig. 4A and B). During the drilling, physiological saline was sprayed on the retractor to prevent skin burn. After the resection, additional curettage and cauterization with an electric scalpel were performed to ensure complete tumor removal (Fig. 4C), although no tumor remnants were detected on CBCT (Fig. 3E). A β-tricalcium phosphate (β-TCP) block (OSferion, Olympus Corp., Tokyo, Japan) was inserted in the bone defect followed by wound disclosure (Figs. 3F, G, and 5 A). A histological evaluation confirmed the diagnosis of osteoid osteoma with differentiating osteoblasts and immature osteoid trabeculae in the nidus surrounded by reactive bones (Fig. 4D and E). Absence of chondrocyte or cartilage formation allowed exclusion of other benign bone lesions such as enchondroma or Bizarre parosteal osteochondromatous proliferation. No atypical cells were found, and malignancies such as osteosarcoma, Ewing’s sarcoma, or adamantinoma were excluded from the differential diagnosis. At the final follow-up 1 yeaar later, the function of the affected limb was normal, without any symptoms. Plain radiography at the final follow-up revealed new bone formation completely filling the bone defect, without local recurrence (Fig. 5B and C).

## 3 Discussion

This case provides two insights into the treatment of osteoid osteoma. A robotic C-arm CBCT imaging system was useful for resecting the tumor, which was not detectable on fluorescence imaging, allowing secure tumor resection with easy and clear CT-like imaging. Use of the dilator and retractor of the MED system and wider trephine tips for bone biopsy made this benign bone tumor surgery less invasive, with only a small skin incision.

Osteoid osteoma is a relatively rare subtype of benign bone tumor, which accounts for 5% of all bone tumors and 11% of benign bone tumors [7]. Osteoid osteoma has a limited likelihood of growth, and tumors ≥1.5 cm are unusual, thus delaying the diagnosis [1,8]. Some patients require additional treatment such as percutaneous ablation [9,10] or surgical excision [4,5] because of resistance to analgesics or persistence of pain.

Thermablation under CT guidance has been reported as a less invasive treatment option [11]. However, its relatively higher recurrence rate ranges from 5% to 20%, and the radiation exposure during CT guidance is an area of concern in this treatment [[12], [13], [14]].

Complete excision of the nidus can cure the benign neoplasm, but intraoperative localization might be difficult even with intraoperative fluoroscopy. The small size of the nidus and extensive sclerosis around it often make it difficult to identify the tumor from the surrounding normal bone on fluorescence. A large amount of bone resection might be required to secure a complete removal, which could lead to bone weakness and fracture [15,16]. A case series of CT-guided open excision surgery for osteoid osteoma reported complete excision with no recurrence, smaller bone defects, and shorter surgical time. Until now, it is considered the most reliable resection method for osteoid osteomas [5].

In the present case, tumor resection under robotic C-arm CBCT guidance for an osteoid osteoma not visible on fluoroscopy was effective in terms of accuracy, less invasiveness, and lower radiation exposure. In recent times, C-arm CBCT has been used in many interventional radiology and oncology procedures, including percutaneous biopsy, ablation, and embolization, as it has advantages to conventional CT [10,17]. C-arm CBCT provides vast cross-sectional tomographic images, so-called CT-like images, to the surgeon intraoperatively by using the C-arm and flat panel detector angiographic systems [10]. The CT-like images can be simultaneously displayed with conventional fluoroscopic images in real time [18]. In addition to the clear intraoperative visualization, C-arm CBCT enables real-time guidance and monitoring of the tumor resection and easy evaluation of post-resection results, and confers lower risk of intraoperative contamination that can occur while moving the patient in and out of the CT scanner and decreased radiation exposure as compared with the conventional CT [9,10]. In the present case, complete tumor resection, as confirmed on post-resection CBCT images, was achieved with minimal sacrifice of the surrounding normal bone under the guidance of C-arm CBCT. Moreover, C-arm CBCT allows optimized planning of the skin entry and a path for less invasive surgery owing to the greater degree of freedom in the robotic C-arm obliquities [9,10]. In the present case, a more minimal invasiveness in terms of the smaller skin and soft-tissue incisions was achieved with the repurposed use of the dilator and retractor of the MED system and wider trephine tips for bone biopsy.

Although there are some limitations of the C-arm CBCT compared to conventional CT, such as a decrease in the number of images taken per image acquisition, increased scatter due to the wider collimation of cone beam, or motion artifacts due to increased acquisition time [19,20], the quality of CBCT images taken in the present case was sufficient for detecting the small nidus.

## 4 Conclusion

In conclusion, C-arm CBCT allowed a clear intraoperative visualization of the osteoid osteoma, precise access to the tumor, accurate tumor resection, and minimal sacrifice of the surrounding normal bone in the present surgery, promising secure resection and a less invasive osteoid osteoma treatment. Furthermore, repurpose use of the dilator and retractor of the MED system, and wider trephine tips for bone biopsy allowed less invasiveness with small skin incision in this case.

## Declaration of Competing Interest

No benefits in any form have been received or will be received from a commercial party related directly or indirectly to the subject of this article.

## Funding

This research did not receive any specific grant from funding agencies in the public, commercial, or not-for-profit sectors.

## Ethical approval

For this type of study, ethics approval was waived by Ethics Committee of our institute.

## Consent

Written informed consent was obtained from the patient’s legal guardian for publication of this case report and any accompanying images.

## Author contribution

Takashi Higuchi: Participation in the surgery, data collection, case analysis, and writing the manuscript. Norio Yamamoto: Participation in the surgery, follow-up the patient, and case analysis. Katuhiro Hayashi: Validation. Akihiko Takeuchi: Validation. Kensaku Abe: Data curation. Yuta Taniguchi: Data curation. Yoshihiro Araki: Data curation. Hiroyuki Tsuchiya: Supervision.

## Registration of research studies

This is not a research study.

## Guarantor

Hiroyuki Tsuchiya, MD, PhD.

## Provenance and peer review

Not commissioned, externally peer-reviewed.

## References

[1] Jaffe H.L. (1953). Osteoid-osteoma. Proc. R. Soc. Med..

[2] Kransdorf M.J., Stull M.A., Gilkey F.W., Moser R.P. (1991). Osteoid osteoma. Radiographics.

[3] Lee E.H., Shafi M., Hui J.H. (2006). Osteoid osteoma: a current review. J. Pediatr. Orthop..

[4] Gibbs C.P., Lewis V.O., Peabody T. (2005). Beyond bone grafting: techniques in the surgical management of benign bone tumors. Instr. Course Lect..

[5] Yang W.-T., Chen W.-M., Wang N.-H., Chen T.-H. (2007). Surgical treatment for osteoid osteoma — experience in both conventional open excision and CT-guided mini-incision surgery. J. Chin. Med. Assoc..

[6] Agha R.A., Borrelli M.R., Farwana R., Koshy K., Fowler A.J., Orgill D.P. (2018). The SCARE 2018 statement: Updating consensus Surgical CAse REport (SCARE) guidelines. Int. J. Surg. (London, England).

[7] Kitsoulis P., Mantellos G., Vlychou M. (2006). Osteoid osteoma. Acta Othop Belg..

[8] Frassica F.J., Waltrip R.L., Sponseller P.D., Ma L.D., McCarthy E.F. (1996). Clinicopathologic features and treatment of osteoid osteoma and osteoblastoma in children and adolescents. Orthop. Clin. North Am..

[9] Cheng E.Y., Naranje S.M., Ritenour E.R. (2014). Radiation dosimetry of intraoperative cone-beam compared with conventional CT for radiofrequency ablation of osteoid osteoma. J. Bone Joint Surg. Am..

[10] Perry B.C., Monroe E.J., McKay T., Kanal K.M., Shivaram G. (2017). Pediatric percutaneous osteoid osteoma ablation: cone-beam CT with fluoroscopic overlay versus conventional CT guidance. Cardiovasc. Intervent. Radiol..

[11] Nijland H., Gerbers J.G., Bulstra S.K., Overbosch J., Stevens M., Jutte P.C. (2017). Evaluation of accuracy and precision of CT-guidance in radiofrequency ablation for osteoid osteoma in 86 patients. PLoS One.

[12] Papathanassiou Z.G., Megas P., Petsas T., Papachristou D.J., Nilas J., Siablis D. (2008). Osteoid osteoma: diagnosis and treatment. Orthopedics.

[13] Rimondi E., Mavrogenis A.F., Rossi G., Ciminari R., Malaguti C., Tranfaglia C., Vanel D., Ruggieri P. (2012). Radiofrequency ablation for non-spinal osteoid osteomas in 557 patients. Eur. Radiol..

[14] Shields D.W., Sohrabi S., Crane E.O., Nicholas C., Mahendra A. (2018). Radiofrequency ablation for osteoid osteoma - recurrence rates and predictive factors. Surgeon.

[15] Cioni R., Armillotta N., Bargellini I., Zampa V., Cappelli C., Vagli P., Boni G., Marchetti S., Consoli V., Bartolozzi C. (2004). CT-guided radiofrequency ablation of osteoid osteoma: long-term results. Eur. Radiol..

[16] Swee R.G., McLeod R.A., Beabout J.W. (1979). Osteoid osteoma. Detection, diagnosis, and localization. Radiology.

[17] Tselikas L., Joskin J., Roquet F., Farouil G., Dreuil S., Hakime A., Teriitehau C., Auperin A., de Baere T., Deschamps F. (2015). Percutaneous bone biopsies: comparison between flat-panel cone-beam CT and CT-scan guidance. Cardiovasc. Intervent. Radiol..

[18] Kroes M.W., Busser W.M., Hoogeveen Y.L., de Lange F., Schultze Kool L.J. (2017). Laser guidance in C-Arm cone-beam CT-guided radiofrequency ablation of osteoid osteoma reduces fluoroscopy time. Cardiovasc. Intervent. Radiol..

[19] Raj S., Irani F.G., Tay K.H., Tan B.S. (2013). C-arm cone beam computed tomography: a new tool in the interventional suite. Ann. Acad. Med. Singapore.

[20] Orth R.C., Wallace M.J., Kuo M.D. (2009). C-arm cone-beam CT: general principles and technical considerations for use in interventional radiology, Journal of vascular and interventional radiology. J. Vasc. Intery. Radiol..

